# Influence of tumor characteristics on correction differences between cone‐beam computed tomography‐guided patient setup strategies in stereotactic body radiation therapy for lung cancer

**DOI:** 10.1111/1759-7714.13261

**Published:** 2019-12-05

**Authors:** Seong Soon Jang, Suk Young Park, Eun Youn Cho, Po Song Yang, Gil Ja Huh, Young Jun Yang

**Affiliations:** ^1^ Department of Radiation Oncology, College of Medicine The Catholic University of Korea Seoul Republic of Korea; ^2^ Department of Internal Medicine Daejeon St. Mary's Hospital Daejeon Republic of Korea; ^3^ Department of Radiation Oncology Daejeon St. Mary's Hospital Daejeon Republic of Korea; ^4^ Department of Radiology, College of Medicine The Catholic University of Korea Seoul Republic of Korea; ^5^ Department of Internal Medicine, College of Medicine The Catholic University of Korea Seoul Republic of Korea

**Keywords:** CBCT, lung cancer, SBRT, setup error

## Abstract

**Background:**

To evaluate the correction differences between vertebra and tumor matching as cone‐beam computed tomography (CBCT)‐guided setup strategies in lung stereotactic body radiation therapy (SBRT), and the correlations with tumor characteristics such as size, mobility, and location.

**Methods:**

The manual registrations for 33 lung tumors treated with SBRT were retrospectively performed by matching thoracic vertebrae for vertebra matching and then by matching CBCT‐visualized tumors within the internal target volume obtained from a four‐dimensional CT dataset for tumor matching.

**Results:**

The mean correction difference between the two matching methods during the SBRT fractions was larger in the anterior‐posterior direction (2.7 mm) than in the superior‐inferior (2.1 mm) and left‐right (1.4 mm) directions, with differences of less than 5 mm in 90% of the total 134 CBCT fractions. The X‐axis and direct distances from the central axis to the tumor had significant correlations with the correction differences in all three directions, while the mobility‐related parameters were correlated only in the superior‐inferior direction. The absolute differences in lung‐dose parameters after applying the margins (3.4–6.5 mm) required for the setup errors from vertebra matching relative to tumor matching were mild, with values of 1.95 Gy for the mean lung dose and 3.9% for V20.

**Conclusion:**

The setup differences between vertebra and tumor matching in the CBCT‐guided setup without rotation correction were increased in tumors located long distances from the central axis. The additional safety margins of 3.4–6.5 mm were required for the setup errors from vertebra matching.

**Key points:**

Significant findings of the study

The correction difference between the vertebra and tumor matching as CBCT‐guided setup strategies was the largest in the anterior‐posterior direction and significantly correlated with the X‐axis and direct distances from the central axis to the tumor.

What this study adds

Setup differences between vertebra and tumor matching in the CBCT‐guided setup were increased in tumors located long distances from the central axis.

The additional safety margins of 3.4–6.5 mm were required for the setup errors from vertebra matching.

## Introduction

Stereotactic body radiotherapy (SBRT) has been widely adopted for medically inoperable early‐stage non‐small cell lung cancer (NSCLC) or metastatic lung cancer and has had a high local control rate and acceptable toxicity in most previously reported studies.^1^, ^2^, ^3^ This approach involves the delivery of an ablative dose to the tumor using highly conformal and oligofractionated (usually less than five fractions) radiation over a short time course. These high doses have been safely delivered with modern conformal techniques by limiting the doses to the surrounding normal tissues. However, uncertainties in patient setup require the need for a safety margin for the target, despite online correction strategies, and uncertainty margins can act as a potential source of severe normal tissue damage by receiving the same ablative doses.^4^

Image guidance is an essential procedure for accurate patient setup and target localization required in SBRT. Online image‐guided radiation therapy (IGRT) verifies the target volume and organ at risk (OAR) locations before daily treatment and can also be used to monitor the target during treatment. With the early use of lung SBRT, orthogonal X‐ray images based on the patient's bony anatomy, which was usually the thoracic vertebrae of the patient as a relatively stable structure, were commonly used by matching with digitally reconstructed radiograph (DRR) images. Later, as cone‐beam computed tomography (CBCT) systems developed, which offer the natural progression from 2D IGRT to 3D volumetric IGRT, the use of 3D volumetric images has been shown to allow a more accurate setup and smaller safety margins than portal imaging with the capability of an additional soft tissue matching with simulation CT to bony structures.^5^ The planning target volume (PTV) is generated by adding a setup margin to the internal target volume (ITV) that is institution‐specific and based on the available image‐guided techniques and a systematic assessment of the positioning reproducibility.^6^ Regarding the size of the setup margin, a uniform expansion of 3 or 5 mm with a CBCT for image guidance during lung SBRT has typically been applied to the ITV to generate the PTV in most institutions.^6^, ^7^, ^8^, ^9^ Furthermore, direct alignment to tumors together with positional correction by 3D vertebra matching is currently preferred as CBCT‐guided setup strategies for improving setup accuracy in lung SBRT.^10^, ^11^ However, to date, reports describing the correction differences between these matching methods, as two commonly employed image guidance structures in CBCT‐guided setup for lung SBRT, have been limited, and the magnitude of these differences based on the tumor characteristics has not been specifically studied.

Therefore, the present study was undertaken to evaluate the correction differences between the vertebra and tumor matching methods as CBCT‐guided setup strategies in lung SBRT and the correlations with tumor characteristics such as size, mobility, and location. In addition, we assessed the required margins in the patient population for setup errors of the vertebra matching method relative to direct tumor matching using a commonly cited margin recipe and the dosimetric consequences for lung OAR by these PTV margin increases.

## Methods

### Patient characteristics

Following the approval of our institutional review board, 33 tumors in 32 patients treated with SBRT for early‐stage NSCLC (29 tumors) or for pulmonary metastases (four tumors) at our institution were included in this retrospective study. The median patient age was 70 years old (range, 48–89 years). The tumor location was the upper lobe for 20 tumors, the middle lobe for two tumors, and the lower lobe for 11 tumors.

### 4D CT acquisition and treatment planning

A four‐dimensional CT (4D CT) technique using a multi‐slice CT scanner (SOMATOM Sensation 64; Siemens Medical Solutions, Erlangen, Germany) was performed for SBRT planning in all patients. The patients were immobilized using a Wing board and a Vac‐Lok body cushion (CIVCO Medical Solutions, Orange City, IA, USA) with their arms placed above their heads. The patients were advised to breathe freely and regularly, and abdominal compression to reduce breathing motion was not applied to any of the patients. A single helical 4D CT scan that included the entire lung was acquired with fixed acquisition parameters (pitch of 0.1, rotation time of 0.5 s, 120 kV, and 400 mA) using a commercially available motion‐monitoring system (AZ‐733V; Anzai Medical, Tokyo, Japan). Using the Syngo software package (Siemens Medical Solutions), the projections were retrospectively sorted based on the corresponding breathing phases (exhalation and inhalation) and the relative amplitudes at 25% intervals from 0% to 100%, and the images were reconstructed into eight respiratory phase bins, which were equally distributed throughout the breathing cycle with a slice thickness of 3.0 mm. Immediately following the 4D CT scan, a modified slow CT scan with the same scanning range and slice thickness was obtained using the same scanner with the longest possible gantry rotation time (1.0 s) and a reduced pitch factor (0.5).

All of the CT datasets were transferred to a commercial treatment‐planning system (Pinnacle^3^ version 8.0m; Philips Medical Systems, Fitchburg, WI, USA), and thereafter, the 4D CT and modified slow CT images were superimposed using an automated algorithm from the Syntegra® software package (Philips Medical Systems). The gross tumor volumes (GTVs) in each of the eight phases of the 4D CT images were delineated with the lung window setting by the same radiation oncologist and were projected onto the modified slow CT image of the same slice. After the ITVs were created by combining the GTVs from all eight phases of the 4D CT, the PTVs were generated by adding a uniform 5‐mm margin to the ITVs without the clinical target volume (CTV) margins. The leaf margin of a 5‐mm was used between the PTV contour and the multileaf collimator. All conformal SBRT plans used 10–14 coplanar and/or noncoplanar beams and were normalized such that at least 95% of the PTV received the prescription dose. The dose‐fractionation schedules were 48 Gy in 4 fractions (20 tumors), 56–60 Gy in 4 fractions (four tumors), and 50–55 Gy in 5 fractions (nine tumors).

Regarding the tumor characteristics, the mean GTV for all eight GTVs and the GTV size at the end of exhalation (EOE) (GTVeoe) were measured as tumor size parameters. Mobility‐related parameters were evaluated with the ITV/GTVeoe ratio, the percentage of overlap volume (POV) between the GTVs from the two extreme bins, and tumor motion in all three directions. The amplitude of the tumor motion was determined by measuring the tumor movement in the eight‐phase 4D CT datasets using the InSpace 4D software package (Siemens Medical Solutions). The motion ranges of the tumor centroid in the superior‐inferior (SI), anterior‐posterior (AP), and left‐right (LR) directions were measured on the transverse, sagittal, and coronal planes using a grid spacing of 1 mm for all eight phase bins registered by this software. Finally, tumor locations were measured as the distances (X‐, Y‐, and Z‐axes and direct distance) from the spinal canal center to the PTV center or margin on the planning image of the PTV center, and the Z‐axis distances from the T1 vertebra and the carina to the PTV center.

### Image guidance using CBCT

A free‐breathing CBCT was acquired prior to each fraction during the SBRT course using a megavoltage (MV)‐CBCT system (MVision; Siemens Medical Solutions) for all patients. This system, with a six MV beam and a 1024 × 1024 amorphous silicon (a‐Si) detector, was used to acquire 200 projections over a 200^o^ arc (270^o^ to 110^o^, clockwise) in a 45‐s interval, with 15 monitor units (MU) delivery for the chest region.^12^ After the acquisition procedure, the CBCT image reconstruction was immediately performed using the protocols for a reconstruction size of 512 × 512, a field size of 27.4 × 27.4 cm^2^, and a slice thickness of 1 mm. The Adaptive Targeting (AT) application of the Coherence Therapist system (Siemens Medical Solutions) was used to retrospectively register the planning CT image dataset with the acquired CBCT image dataset, which had previously been stored with isocenter and contour information after CBCT‐guided corrections. After autoregistration of the two image datasets using the mutual information algorithm in the AT software, a radiation oncologist performed the manual registrations by the matching of thoracic vertebrae and spinal canal contours in three planes for the vertebra matching and then by matching the visualized target volume on the CBCT image within the ITV on the planning CT in three planes for the direct tumor matching. The displayed table offset values for correction in the AT software were recorded in the LR, SI, and AP directions after each matching.

### Margin calculation and dosimetric analysis

The required margins in the patient population for setup errors of the vertebra matching method relative to the tumor matching were calculated using the margin formula (M = 2.5∑_p_ + 0.7σ_p_) described by van Herk *et al*.^13^, ^14^ Briefly, the PTV margin (M), which had been defined as the margin required to ensure a minimum clinical target volume (CTV) dose of at least 95% of the prescription dose for 90% of the patients in their margin recipe, was calculated in the three directions from the values of systematic error (∑_p_) and random error (σ_p_) in the population. The group mean (GM) was the average of all individual mean errors over the treatment course. The population systematic error (∑_p_) was the standard deviation (SD) of the individual means as a measure of the interpatient variability. The population random error (σ_p_) was defined as the root‐mean‐square of the individual SDs, which was indicative of the intrapatient variability.

To assess the dosimetric impact of the PTV increase on normal lung tissue, another PTV (PTV_ReqM_) was generated by adding these required margins in the three directions to our conventional PTV (PTV_Conv_), which has a 5 mm isotropic margin from the ITV. Two conformal SBRT plans for all 33 tumors were performed using the two PTVs (PTV_Conv_ and PTV_ReqM_). For the dose‐fractionation schedules of 48–60 Gy in 4–5 fractions, the beam energies, weights, and gantry angles remained fixed for each tumor in the same beam configuration that was used for the actual patient treatment to allow for meaningful comparisons. To ensure a more realistic lung volume during treatment, the dose distributions were calculated on the modified slow CT images for the two PTVs, with heterogeneity corrections applied using the collapsed cone convolution superposition algorithm. The dosimetric effects on a normal lung of SBRT planning using the two different PTVs were analyzed via lung‐dose parameters, such as the mean lung dose (MLD) and the percentage volumes of both lungs minus the ITV receiving specific doses of 5, 10, 20, 25, and 30 Gy (V5, V10, V20, V25, and V30) according to dose‐volume histogram estimations.

### Statistical analysis

The correlations between the correction differences and the tumor parameters for each tumor were evaluated using Pearson correlation analyses. Additionally, the correction differences between the tumor parameter groups were assessed using the Mann‐Whitney test. To compare dosimetric differences between each pair of lung‐dose parameters, we used paired *t*‐tests for each tumor. All statistical analyses were performed using the SPSS software package (version 15.0; SPSS Inc., Chicago, IL, USA). Values of *P* < 0.05 were considered to be significant.

## Results

### Tumor characteristics

The characteristics of the 33 tumors, including the size, mobility, and location, are outlined in Table 1. The mean of the GTVs from all eight phases in all tumors was 9.8 ± 9.0 cc (range: 0.7–35.0 cc). The tumor motions were most extensive in the SI direction (6.5 ± 4.7 mm) and were approximately 1.4–1.9 times greater in this direction than in the AP (4.5 ± 3.5 mm) and LR (3.5 ± 3.1 mm) directions. The mean 3D mobility for all 33 tumors, which was calculated as (SI^2^ + AP^2^+LR^2^)^1/2^, was 8.9 ± 6.3 mm. The ITV/GTVeoe ratio and the POV between the GTVs from the 2 extreme bins measured as the mobility‐related parameters were 1.65 ± 0.35 and 55.8 ± 22.2%, respectively. Regarding the tumor location, the direct distance from the spinal canal center to the PTV center was 8.72 ± 2.53 cm, and their distances on the X‐ and Y‐axes were 7.27 ± 1.78 cm and 3.93 ± 3.33 cm, respectively. The Z‐axis distances from the T1 vertebra and the carina to the PTV center were 11.72 ± 4.01 cm and 3.78 ± 2.80 cm, respectively.

**Table 1:** Tumor characteristics (*n* = 33)

Tumor parameters	Mean ± SD	Range
Size		
Mean GTV (cc)	9.8 ± 9.0	0.7–35.0
GTVeoe (cc)	9.4 ± 8.7	0.7–32.8
Mobility		
ITV/GTVeoe ratio	1.65 ± 0.35	1.23–2.42
POV between GTVs from 2 extreme bins (%)	55.8 ± 22.2	11.9–85.1
SI motion (mm)	6.5 ± 4.7	1.0–25.0
AP motion (mm)	4.5 ± 3.5	0.5–17.0
LR motion (mm)	3.5 ± 3.1	0.5–15.0
3D mobility (mm)	8.9 ± 6.3	1.2–33.8
Location		
X‐axis distance from SC center to PTV center (cm)	7.27 ± 1.78	3.01–10.19
Y‐axis distance from SC center to PTV center (cm)	3.93 ± 3.33	0.12–12.03
Direct distance from SC center to PTV center (cm)	8.72 ± 2.53	5.27–15.28
Z‐axis distance from SC center to PTV margin (cm)	2.16 ± 0.61	1.03–3.56
Z‐axis distance from T1 vertebra to PTV center (cm)	11.72 ± 4.01	5.55–21.00
Z‐axis distance from carina to PTV center (cm)	3.78 ± 2.80	0.15–10.95

GTV, gross tumor volume; GTVeoe, GTV at the end of exhalation; ITV, internal target volume; POV, percentage of overlap volume; PTV, planning target volume; SC, spinal canal; SD, standard deviation; SI, superior‐inferior; AP, anterior‐posterior; LR, left‐right directions.

### Setup differences between the vertebra and tumor matching and correlations with tumor characteristics

**Figure 1:**
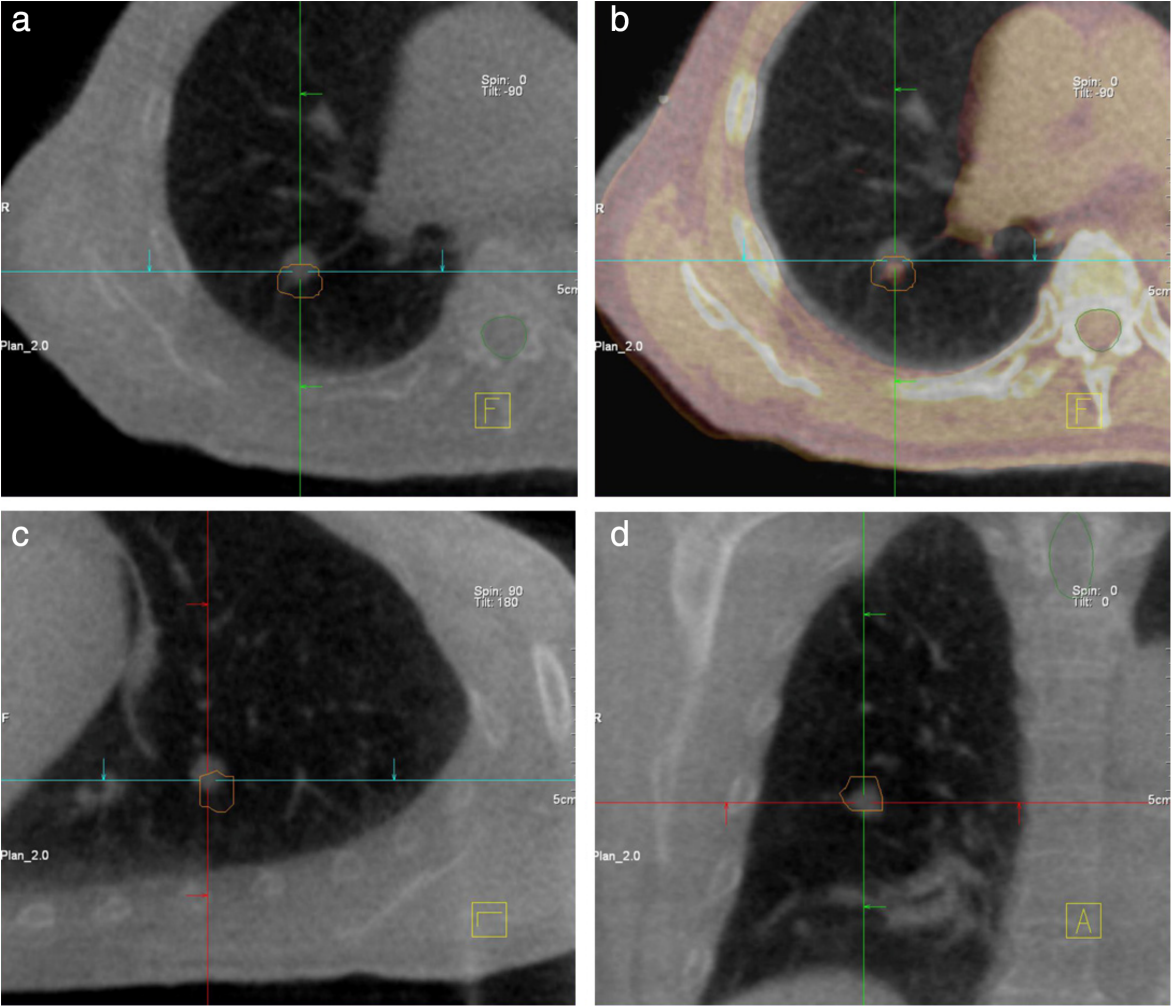
Example of the correction mismatch between the vertebra and tumor matching. There was a considerable mismatch between the cone‐beam computed tomography (CBCT)‐visualized tumor and the internal target volume (ITV, orange color) of the planning CT with a rotational setup error (**b**), despite accurate vertebra matching. (**a** and **b**) Transverse view; (**c** and **d**) sagittal (**c**) and coronal (**d**) views.

For all 33 tumors, the maximum correction difference between the two matching methods during all 4–5 treatment fractions was the largest in the AP direction (4.4 ± 3.1 mm), and the mean difference was also larger in this direction (2.7 ± 2.1 mm) than in the SI (2.1 ± 1.7 mm) and LR (1.4 ± 1.0 mm) directions. Regarding the 3D values for the three directions, the maximum and mean differences during the treatment fractions were 6.2 ± 3.5 mm and 4.3 ± 2.5 mm, respectively (Table 2, Fig 1). In addition, 32.1% and 9.7% of the total 134 CBCT fractions in the AP direction exhibited differences exceeding 3 and 5 mm, respectively, and 90% of the total fractions had differences of less than 5 mm in the three directions (Table 3).

**Table 2:** Setup correction differences between the vertebra and tumor matching methods during all 4–5 treatment fractions (*n* = 33)

Directions	Maximum difference (mm) (mean ± SD, range)	Mean difference (mm) (mean ± SD, range)
LR	2.7 ± 2.0 (1.0–10.0)	1.4 ± 1.0 (0.2–4.3)
SI	3.5 ± 2.2 (0–10.0)	2.1 ± 1.7 (0–7.5)
AP	4.4 ± 3.1 (0–12.0)	2.7 ± 2.1 (0–8.0)
3D	6.2 ± 3.5 (1.0–16.1)	4.3 ± 2.5 (0.3–10.9)

LR, left‐right; SI, superior‐inferior; AP, anterior‐posterior directions; SD, standard deviation.

**Table 3:** Setup correction differences between the vertebra and tumor matching in a total of 134 CBCT fractions

Directions	Mean ± SD (mm)	Maximum (mm)	90th percentile (mm)	>3 mm (fractions, %)	>5 mm (fractions, %)
LR	1.4 ± 1.5	10.0	3.0	10 (7.5%)	2 (1.5%)
SI	1.9 ± 2.0	10.0	4.0	21 (15.7%)	8 (6.0%)
AP	2.6 ± 2.5	12.0	5.0	43 (32.1%)	13 (9.7%)
3D	4.1 ± 2.8	16.1	8.1	—	—

CBCT, cone‐beam computed tomography; LR; SI; AP, left‐right; superior‐inferior; anterior‐posterior directions; SD, standard deviation.

**Figure 2:**
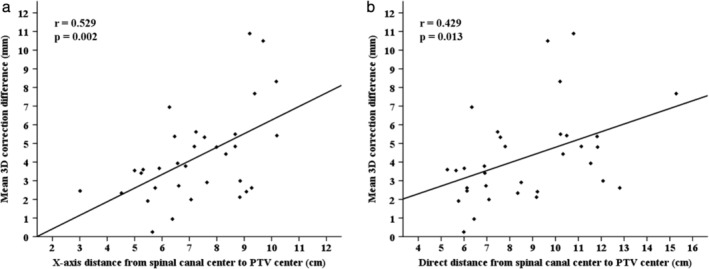
Mean correction difference (3D) between the vertebra and tumor matching as a function of the distance from the central axis to the tumor. Here, r represents the correlation coefficient.

Regarding the correlations (Table 4) between the correction differences and tumor characteristics, tumor size parameters did not exhibit significant correlations with the correction differences in any direction. All mobility‐related parameters were significantly correlated with the maximum and mean differences only in the SI direction and therefore with the mean 3D difference. Interestingly, the X‐axis and direct distances from the spinal canal center to the PTV center were revealed to be more powerful parameters by significant correlations with the maximum and/or mean differences in all three directions and the 3D differences (Table 4, Fig 2). In addition, when the 3D correction differences between tumor groups using cutoff values of 8 cm for the X‐axis distance and 10 cm for the direct distance were compared, significant differences between two groups were shown, with values of 3.3–3.6 mm in maximum difference and 2.1 mm in mean difference (Table 5). The Y‐axis distance and the Z‐axis distances correlated with the correction differences only in the LR direction and only in the SI direction, respectively. The correction differences between the tumors of the upper/middle lobe and lower lobe locations were not significantly different (6.2 ± 3.1 and 4.1 ± 1.8 mm vs. 6.2 ± 4.3 and 4.7 ± 3.5 mm in the 3D maximum and mean differences, *P* > 0.05).

**Table 4:** Correlations between the correction differences and tumor parameters

Tumor parameters	Correction differences with significant correlations (*r*‐value, *P*‐value: *P < 0.05 and **P < 0.01)
Mean GTV	NS
GTVeoe	NS
ITV/GTVeoe ratio	Max SI (0.375, *), Mean SI (0.418, *)
POV between GTVs from two extreme bins	Max SI (−0.491, **), Mean SI (−0.469, **), Mean 3D (−0.349, *)
SI motion	Max SI (0.593, **), Mean SI (0.561, **), Mean 3D (0.420, *)
AP motion	Max SI (0.537, **), Mean SI (0.532, **), Mean 3D (0.412, *)
LR motion	Max SI (0.529, **), Mean SI (0.566, **), Mean 3D (0.432, *)
3D mobility	Max SI (0.582, **), Mean SI (0.569, **), Mean 3D (0.423, *)
X‐axis distance from SC center to PTV center	Mean LR (0.403, *), Max SI (0.460, **), Mean SI (0.439, *), Max AP (0.419, *), Mean AP (0.413, *), Max 3D (0.492, **), Mean 3D (0.529, **)
Y‐axis distance from SC center to PTV center	Max LR (0.449, **), Mean LR (0.423, *)
Direct distance from SC center to PTV center	Max LR (0.542, **), Mean LR (0.603, **), Max SI (0.409, *), Mean SI (0.366, *), Max AP (0.342, *P* = 0.05), Max 3D (0.542, **), Mean 3D (0.429, *)
Z‐axis distance from SC center to PTV margin	Max SI (0.413, *), Mean SI (0.494, **)
Z‐axis distance from T1 vertebra to PTV center	Mean SI (0.380, *)
Z‐axis distance from carina to PTV center	Mean SI (0.344, *P* = 0.05)

GTV, gross tumor volume; GTVeoe, GTV at the end of exhalation; ITV, internal target volume; Max and Mean SI/LR/AP/3D, maximum and mean correction differences in the SI/LR/AP/3D directions during the treatment fractions; NS, no significant; POV, percentage of overlap volume; PTV, planning target volume; r, Pearson correlation coefficient; SC, spinal canal; SI; AP; LR, superior‐inferior; anterior‐posterior; left‐right directions.

**Table 5:** Comparisons of the 3D correction differences based on the distance from the spinal canal center to the PTV center

Tumor parameter groups	Max 3D difference (Mean ± SD, mm)	Mean 3D difference (Mean ± SD, mm)
X‐axis distance from SC center to PTV center		
≤8 cm (*n* = 21)	5.0 ± 2.4	3.5 ± 1.6
>8 cm (*n* = 12)	8.3 ± 4.2 (*P* = 0.026)	5.6 ± 3.1 (*P* = 0.047)
Direct distance from SC center to PTV center		
≤10 cm (*n* = 21)	4.9 ± 2.6	3.5 ± 2.2
>10 cm (*n* = 12)	8.5 ± 3.8 (*P* = 0.003)	5.6 ± 2.4 (*P* = 0.006)

Max, maximum; PTV, planning target volume; SC, spinal canal; SD, standard deviation.

### Calculated safety margin and dosimetric analysis

From the systematic and random error data of corrections by vertebra matching relative to the tumor matching method, we calculated the required PTV margin in the whole patient population using the van Herk margin recipe (Table 6). The population systematic errors of the vertebra matching method were 1.0, 1.7, and 2.1 mm in the LR, SI, and AP directions, respectively, with the same order for the size of the random errors. Therefore, the size of the required margins in the SI (5.4 mm) and AP (6.5 mm) directions were larger than that in the LR (3.4 mm) direction. Additionally, the size of these margins was larger in the patient population with tumors (*n* = 12) located at distances more than 8 cm (X‐axis distance) or 10 cm (direct distance) from the spinal canal center, with values of 1.2‐ to 2.7‐fold in the three directions, based on the results of increased systematic and random errors in these groups (Table 6).

**Table 6:** Setup errors of the vertebra matching and the required margins based on the patient population

Patient populations	LR (mm)	SI (mm)	AP (mm)
Whole population (*n* = 33)			
GM	1.4	2.1	2.7
∑_p_	1.0	1.7	2.1
σ_p_	1.4	1.6	1.8
M	3.4	5.4	6.5
X‐axis distance from SC center to PTV center ≤8 cm (*n* = 21)/>8 cm (*n* = 12)			
GM	1.2/1.9	1.5/3.0	2.3/3.5
∑_p_	0.8/1.0	0.8/2.5	1.7/2.6
σ_p_	1.2/1.7	1.3/2.0	1.3/2.5
M	2.9/3.7	2.8/7.5	5.1/8.3
Direct distance from SC center to PTV center ≤10 cm (*n* = 21)/>10 cm (*n* = 12)			
GM	1.1/2.0	1.5/3.0	2.4/3.3
∑_p_	0.6/1.2	1.1/2.2	2.1/2.1
σ_p_	1.0/1.8	1.3/1.9	1.2/2.5
M	2.3/4.2	3.6/6.9	6.1/7.1

∑_p_, systematic error in population; σ_p_, random error in population; GM, group mean; LR; SI; AP, left‐right; superior‐inferior; anterior‐posterior directions; M, the calculated margin using the van Herk formula (M = 2.5∑_p_ + 0.7σ_p_); PTV, planning target volume; SC, spinal canal.

The comparisons of the lung‐dose parameters for the two SBRT plans based on the different PTV definitions (PTV_Conv_ and PTV_ReqM_) in all 33 tumors are summarized in Table 7. Although the PTV increase (40.0 ± 25.6 *vs*. 84.1 ± 43.7 cc) after applying these additional margins was considerable at 2.3‐fold, the magnitude of the absolute differences between the two plans in the lung‐dose parameters was small. The average dose increases in the normal lung by these PTV increases were 1.95 Gy for MLD and 3.9% for V20.

**Table 7:** Absolute differences in the lung‐dose parameters for the 2 SBRT plans (*n* = 33) based on the different PTV definitions

			PTV_Conv_ *vs*. PTV_ReqM_	
Parameters	PTV_Conv_ (Mean ± SD)	PTV_ReqM_ (Mean ± SD)	Mean (range)	*P*
MLD (Gy)	4.86 ± 1.61	6.81 ± 2.11	1.95 (1.26–3.77)	0.000
V5 (%)	20.52 ± 5.90	26.79 ± 7.62	6.27 (3.03–11.90)	0.000
V10 (%)	14.53 ± 4.41	19.36 ± 5.46	4.83 (3.03–8.97)	0.000
V20 (%)	7.61 ± 3.43	11.48 ± 4.31	3.86 (1.96–7.40)	0.000
V25 (%)	5.64 ± 2.81	9.01 ± 3.84	3.37 (1.79–6.72)	0.000
V30 (%)	4.26 ± 2.21	7.07 ± 3.15	2.81 (1.51–5.55)	0.000

MLD, mean lung dose; PTV, planning target volume; SBRT, stereotactic body radiation therapy; SD, standard deviation; V5, V10, V20, V25, and V30, percentage volumes of both lungs minus the internal target volumes receiving specific doses of 5, 10, 20, 25, and 30 Gy.

## Discussion

The uncertainties in lung SBRT can occur at several points of target delineation, daily patient setup, and motion management and a reduced safety margin through minimization of these uncertainties is required, due to the high fractional dose.^4^ For lung SBRT, motion management and IGRT are the recommended standard of care.^5^ We mainly focused on the CBCT‐guided strategies for accurate patient setup in this study because 4D CT information is already used as an important tool for motion management. At our institution, a PTV for lung SBRT planning is typically constructed by adding a 5 mm setup margin to the ITV in consideration of some residual or intrafractional setup error. In the practice of CBCT‐guided daily setup, the treating physician performs a manual registration procedure through the matching of thoracic vertebra and spinal canal structures after checking the fusion accuracy of the autoregistration between the acquired CBCT and the planning CT using the AT software in the treatment workstation. Then, the physician determines the final couch shifts in three translational directions to the treatment position with confirmation of the adequate inclusion of the visualized tumor on the CBCT image within the PTV on the planning CT or, if there is no adequate inclusion, with additional manual registration by direct tumor matching within the center of the PTV. Because the acquisition spans several respiratory cycles over a long period of time, the free‐breathing CBCT image should generate an ITV that captures the full range of motion and represents the time‐averaged position of the target.^15^ Moreover, the direct tumor matching strategy using a relatively well visible tumor on CBCT images compared to the other sites, where the indirect alignment based on nearby anatomy or fiducial markers is mostly used, would be especially useful in lung SBRT. In this study, we used the ITV instead of the PTV used in our practice as a target volume for tumor matching on the planning CT for a more accurate comparison between the two matching methods. In another study comparing the setup differences using the vertebra and tumor as two important image guidance structures in lung SBRT, Corradetti *et al*.^16^ reported that setup errors of bony anatomy matching using orthogonal X‐ray imaging in lung cancer patients treated with SBRT, which were measured by tumor matching in pretreatment CBCT, exhibited errors of 1.5, 1.8, and 2.2 mm in LR, SI, and AP directions, respectively. However, despite these similar results to our mean values of differences between the two matching methods on the 3D‐CBCT, their systematic (1.5–2.7 mm) and random (1.7–2.7 mm) errors were larger than ours (1.0–2.1 mm for ∑_p_ and 1.4–1.8 mm for σ_p_), and therefore had larger values (4.9–8.5 mm) in the margins calculated by the van Herk formula. Furthermore, in the Worm *et al*. study^17^ with 3D‐CBCT, the 3D baseline shift (3.9 ± 2.0 mm) for 18 lung tumors, which was defined as the difference between the bony anatomy matching using vertebra spine and the tumor matching to planning GTV + 1 cm margin without 4D CT information, was similar to our mean 3D difference (4.3 ± 2.5 mm). However, their systematic errors were also quite large, with values of 2.9 mm in the SI and AP directions. Taken together with these similar results in the magnitude of setup differences, the use of 3D image guidance and 4D CT information in daily patient setup may be methods for preventing large safety margins by decreasing inter‐ and/or intrapatient variability.

The possible causes of the setup differences between these two matching methods were analyzed in terms of correlations with the tumor characteristics. In our results, the mobility‐ and Z‐axis distance‐related parameters were correlated with the setup difference only in the SI direction. Some studies have reported that the ITVs generated from such a CBCT scan could underestimate the target volume because of disparities in breathing or image quality depending on the target location.^15^, ^18^ This correlation may be attributable to an inaccurate motion range in the SI direction, which exhibited more extensive mobility than the range in the other directions, on the CBCT image. As more powerful parameters, the X‐axis and direct distances from the central axis to the tumor had significant correlations with the setup differences in all three directions. A possible explanation could be the increasing likelihood of a mismatch between the two matching methods by a rotational setup error in the tumors located at long distances from the central axis with only a translational correction.

For our whole population, the required PTV margins from the setup errors of the vertebra matching relative to the tumor matching progressively increased from the LR to the SI to the AP direction. In the AP direction with the largest mean setup difference between the two matching methods, a larger margin (6.5 mm) was required than those in the SI (5.4 mm) and LR (3.4 mm) directions due to the larger errors in this direction. As the margin required to ensure an adequate CTV dose in 90% of the population, the importance of systematic error rather than random error is emphasized by van Herk.^13^ The size of systematic errors in the SI and AP directions were 1.7‐ to 2.1‐fold larger than that in the LR direction, whereas these values were 1.1‐ to 1.3‐fold for the random error, and therefore the larger errors in these directions led to larger margins. As the 3D total vector shifts, our margins in three directions corresponded to an isotropic margin of approximately 5.3 mm. Together with this margin result, the results of the setup difference in the total 134 CBCT fractions, which exhibited a difference of less than 5 mm in all three directions in 90% of the total fractions, suggested that the setup errors from vertebra matching relative to tumor matching require an additional safety margin of approximately 5 mm. The size of this suggested margin could be useful as a reference in conditions with only a positional setup by vertebra‐based matching and without either direct tumor alignment in CBCT‐guided setup procedure or the capacity for tumor matching due to a poor quality tumor image on the CBCT. However, caution is advised for the tumor population located far from the central axis because large margins of approximately 7–8 mm in the SI and AP directions were needed in this study. In addition, due to the center‐ and procedure‐specific natures of these margins from setup errors, the margin needs to be specifically evaluated for the SBRT procedure and equipment in a particular institution.^4^

Regarding the dosimetric analysis of the normal lung, although by applying these additional margins the PTV increase was quite large, the absolute differences in lung‐dose parameters were mild. These results may have been due to the highly conformal SBRT plans for small target volumes. From the clinically recommended dose constraints and the results in lung SBRT for the risk of radiation pneumonitis,^19^, ^20^, ^21^, ^22^ the benefit of a proper safety margin for preventing a marginal miss of the tumor should also be considered relative to the low risk of toxicity under these lung doses.

In conclusion, the mean correction difference between the vertebra and tumor matching methods during the SBRT fractions in the CBCT‐guided setup was the largest in the AP direction (2.7 mm) compared to the SI (2.1 mm) and LR (1.4 mm) directions and had a difference of less than 5 mm in 90% of total CBCT fractions. The X‐axis and direct distances from the central axis to the tumor had significant correlations with the correction differences in all three directions in the setup without rotational error correction. The setup errors from vertebra matching relative to tumor matching required the additional safety margins of 3.4–6.5 mm, and the absolute differences in lung‐dose parameters after applying these margins were mild.

## Disclosure

None of the authors reports any conflicts of interest.
